# Determining wave direction using curvature parameters

**DOI:** 10.1016/j.mex.2016.01.003

**Published:** 2016-01-16

**Authors:** Eduardo Vitarelli de Queiroz, João Luiz Baptista de Carvalho

**Affiliations:** Laboratory of Physical Oceanography, University of Vale do Itajaí, Rua Uruguai, 458, Itajaí, SC 88.302-202, Brazil

**Keywords:** Waves direction, Curvature parameter, Slope parameter, Sea level, Numerical simulation, Parametrical estimation

## Abstract

The curvature of the sea wave was tested as a parameter for estimating wave direction in the search for better results in estimates of wave direction in shallow waters, where waves of different sizes, frequencies and directions intersect and it is difficult to characterize. We used numerical simulations of the sea surface to determine wave direction calculated from the curvature of the waves. Using 1000 numerical simulations, the statistical variability of the wave direction was determined. The results showed good performance by the curvature parameter for estimating wave direction. Accuracy in the estimates was improved by including wave slope parameters in addition to curvature. The results indicate that the curvature is a promising technique to estimate wave directions.

• In this study, the accuracy and precision of curvature parameters to measure wave direction are analyzed using a model simulation that generates 1000 wave records with directional resolution.
• The model allows the simultaneous simulation of time-series wave properties such as sea surface elevation, slope and curvature and they were used to analyze the variability of estimated directions.
• The simultaneous acquisition of slope and curvature parameters can contribute to estimates wave direction, thus increasing accuracy and precision of results.

## Graphical abstract

**Figure:**
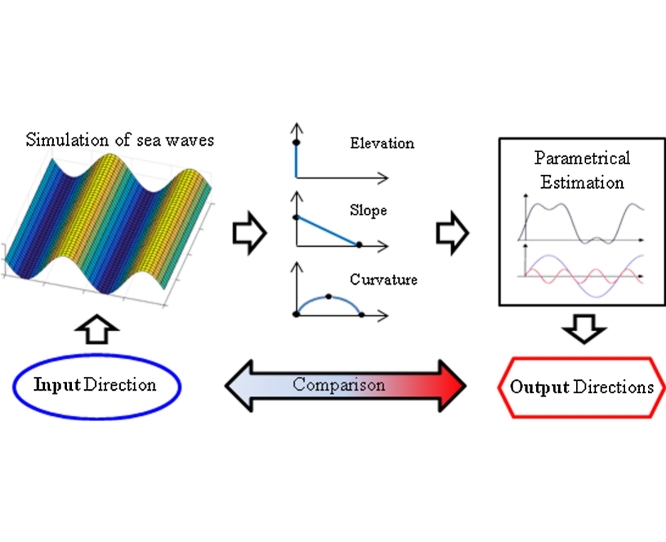

## Method details

### Numerical simulation

In this study, the method of wave simulation recording proposed by Goda [1], with directional resolution and based on trigonometric functions, was used. The method allows the simultaneous simulation of wave properties such as elevation (*η*), slope (*η*_*x*_, *η*_*y*_) and curvature (*η*_*xx*_, *η*_*yy*_) of the sea surface level (Eqs. (1), (6), (7), (8), (9), (10), (11), (12), (13), respectively)

(1) $$\eta (x,y,t)=\sum_{m=1}^{M}\sum_{N=1}^{N}a_{m,n}\cos[k_{m}x\cos\;\theta_{n}+k_{m}y\sin\;\theta_{n}-2\pi f_{m}t+\varepsilon_{m,n}]$$

(2) $$\eta_{x}(x,y,t)=\sum_{m=1}^{M}\sum_{N=1}^{N}a_{m,n}[-k\cos\;\theta_{n}]\sin[k_{m}x\cos\;\theta_{n}+k_{m}y\sin\;\theta_{n}-2\pi f_{m}t+\varepsilon_{m,n}]$$

(3) $$\eta_{y}(x,y,t)=\sum_{m=1}^{M}\sum_{N=1}^{N}a_{m,n}[-k\sin\;\theta_{n}]\sin[k_{m}x\cos\;\theta_{n}+k_{m}y\sin\;\theta_{n}-2\pi f_{m}t+\varepsilon_{m,n}]$$

(4) $$\eta_{xx}(x,y,t)=\sum_{m=1}^{M}\sum_{N=1}^{N}a_{m,n}{\left[-k\cos\;\theta_{n}\right]}^{2}\cos[k_{m}x\cos\;\theta_{n}+k_{m}y\sin\;\theta_{n}-2\pi f_{m}t+\varepsilon_{m,n}]$$

(5) $$\eta_{yy}(x,y,t)=\sum_{m=1}^{M}\sum_{N=1}^{N}a_{m,n}{\left[-k\sin\;\theta_{n}\right]}^{2}\cos[k_{m}x\cos\;\theta_{n}+k_{m}y\sin\;\theta_{n}-2\pi f_{m}t+\varepsilon_{m,n}]$$

where *θ* is the wave direction, *f* is the frequency and *ɛ* phase. *M* and *N* are respectively the numbers of frequency and direction components. The random phase term (*ɛ*_*m,n*_) has to be distributed between 0 and 2π. The wave number (*k*) has to satisfy the dispersion relation:

(6) $$4\pi^{2}f_{m}^{2}=gk\tan\;h(kh)$$

where *g* is the gravity acceleration and *h* is the local deep.

The term *a*_*m,n*_ (Eqs. (1), (6), (7), (8), (9), (10), (11), (12), (13)) is the amplitude of the wave with *m* frequency and *n* direction and it is calculated by directional spectrum:

(7) $$a_{m,n}=2\sqrt{S(f_{m},\theta_{n})\Delta f_{m}\Delta \theta_{n}}$$

The sampling rate (Δ*t* = *t*_*i+*1_ − *t*_*i*_) is usually constant in a simulated record wave. Thus the trigonometric functions can be obtained with *t = t*_*i*_, using trigonometric relationships additions:

(8) $$\cos(2\pi f_{m}t_{i+1})=\cos(2\pi f_{m}t_{i})\cos(2\pi f_{m}\Delta t)-\sin(2\pi f_{m}t_{i})\sin(2\pi f_{m}\Delta t)$$

(9) $$\sin(2\pi f_{m}t_{i+1})=\sin(2\pi f_{m}t_{i})\cos(2\pi f_{m}\Delta t)+\cos(2\pi f_{m}t_{i})\sin(2\pi f_{m}\Delta t)$$

The frequency components must be independent of each other as discussed by [2]. This effect can be obtained by *f*_*m*_ = *f*_*m−*1_* + rand* Δ*f*. The term rand is a representation of a random number generating function.

The JONSWAP spectral model was developed by Hasselmann et al. [3] and it is an appropriate model to simulate wave spectra in shallow waters because it is applied for developing seas with limited space. Goda [1] showed a variation of the spectrum with three dependent parameters (*α*, *γ* and *σ*). This model allows simulating sea conditions and it is adjusted by function:

(10) $$S(f)=\alpha_{0}H_{1/3}^{2}f_{m}^{4}f^{-5}\exp[-1.25{\left(f_{m}/f\right)}^{4}]\gamma^{\exp[-{\left(f/{f_{m}}^{-1}\right)}^{2}/2\sigma^{2}]}$$

where $\alpha_{0}=\frac{0.0624}{0.23+0.0336\gamma -0.185{\left(1.9+\gamma \right)}^{-1}}$

We used mean values for the following parameters [3]:

*α* is the scale parameter ($0.076{\bar{x}}^{-0.22}$), where $\bar{x}$ is the dimensionless fetch (*gx*/*U*^2^), *x* is the dimensionless fetch length and *U* is the wind speed.
*γ* is the shape parameter (3.30) and *f*_*m*_ is the peak frequency $\left(3.5\frac{g}{U}{\bar{x}}^{-0.33}\right)$.
*σ* is the width of spectral peak $\left\{\begin{array}{c} \sigma_{a}=0.07\;\text{for}\;f\leq f_{m}\\ \sigma_{b}=0.09\;\text{for}\;f>f_{m} \end{array}\right)$

The model simulation is capable of reproducing the statistical variability of wave parameters as it occurs in nature, even in fields considered homogeneous. As it shows normal distribution, the parameters extracted from the simulation obey the central limit theorem. Therefore, the results obtained from a simple execution of the simulation software should not be taken as conclusive. Rather, many simulations are required to ensure that the distribution of the parameter reaches the limit [4].

The wave records were simulated using the data input showed in Table 1. The direction of waves estimated from the parametrization of slope and curvature of waves was compared with the data input properties of the model. One thousand (1000) wave records were generated.

### The parametrical estimation method

The formulation needed to obtain the directional wave spectrum was shown by Longuet-Higgins et al. [5] and Borgman [2]. The formulation begins with a system of integral equations obtained from the function of space-temporal covariance between two independent properties of the wave. We used three parameters (Elevation (*η*), slope (*η*_*y*_, *η*_*xx*_) and curvature (*η*_*xx*_, *η*_*yy*_). Their solution provides the directional spectral density for each frequency. Applying the directional spectral density and angular spreading function (expanded as a Fourier series) to the system of integral equations reduces them to an easily solved linear system. The unknown factors then become the Fourier coefficients, which are used to reconstitute the waves direction. The cross and quadrature spectral densities are used to calculate the Fourier coefficients and come from the Fast Fourier Transform of the cross-correlation between the parameters *η*, *η*_*x*_, *η*_*y*_, *η*_*xx*_ and *η*_*yy*_.

The curvature of the wave allows to calculation of two pairs of first order Fourier coefficients of (*a*_1_ and *b*_1_) and four second order coefficients (*a*_2_). The first and second order Fourier coefficients are determined from the equations showed in Table 2.

The mean direction (Eq. (11)) is given by the first-order component (*a*_1_ and *b*_1_) and the second-order coefficients (*a*_2_ and *b*_2_) are used to measure the principal direction Eq. (12):

(11) $$\theta_{0}=\arctan\left(\frac{b_{1}}{a_{1}}\right)$$

(12) $$\theta_{p}=\frac{1}{2}\arctan\left(\frac{b_{2}}{a_{2}}\right)$$

### Check ratio

The Check ratio, with the ideal result of 1, was used to test the efficiency of the parameters. The Check ratio (*k*/*k*_*c*_) uses the vertical and horizontal parameters of the waves (Eq. (13)) to estimate the wave number (*k*_*c*_) and may be compared to the number of wave number (*k*) obtained from the dispersion relation based on linear theory (Eq. (6))

The wave number (*k*_*c*_) is determined by:

(13) $$k_{c}={\left(\frac{S\eta_{x}\eta_{x}(f)+S\eta_{y}\eta_{y}(f)}{S\eta \eta (f)}\right)}^{1/2}$$

### Accuracy and precision of the system

The first (*a*_1_ and *b*_1_) and second (*a*_2_ and *b*_2_) orders Fourier coefficients were obtained from the elevation, slope and curvature parameters (Table 2). The accuracy of the method was tested by comparing the results of mean directional wave records generated in seven different directions within the quadrant 0–90°. The precision was calculated as variation (standard deviation) of the direction estimates at the peak frequency of energy from 1000 simulated records. All the analysis have been written in Matlab software.

## Results

**Fig. 1:**
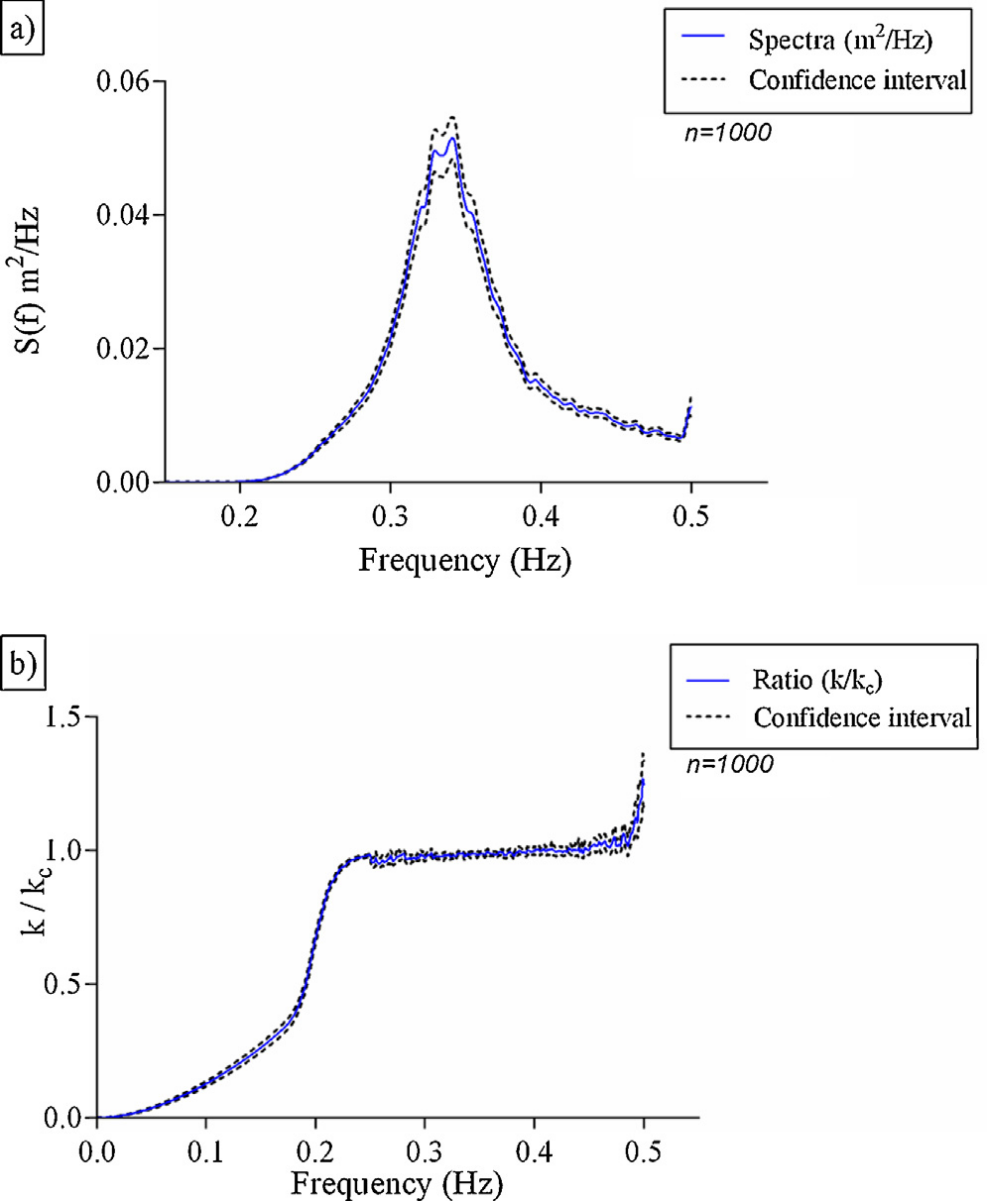
Power spectrum (a), Check ratio, (b) in a 99% confidence interval.

The results showed that the spectral model presents a realistic simulation of developing sea with limited space, which are characteristics of shallow waters. As expected, a narrow confidence interval (99%) was observed when a large number of simulations were used, and a wider one was observed in the higher frequency of the power spectrum (Fig. 1a). The power spectrum showed a frequency of energy band from 0.2 to 0.5 Hz, with a peak frequency of energy (mean of 0.05 m^2^/Hz) at 0.33 Hz. The results were similar to the model data input that showed a peak period of 3 s and significant height of 0.3 m (Table 1).

The values of wave number obtained from wave parameters of elevation and slope (Eq. (14)) and from the dispersion relation (Eq. (6)) showed the trueness of the simulation in all energy frequency bands (Fig. 1b). The mean ratios (*k*/*k*_*c*_) and the confidence interval (99%) calculated on the peak frequency of energy were 1 and 0.01, respectively.

**Fig. 2:**
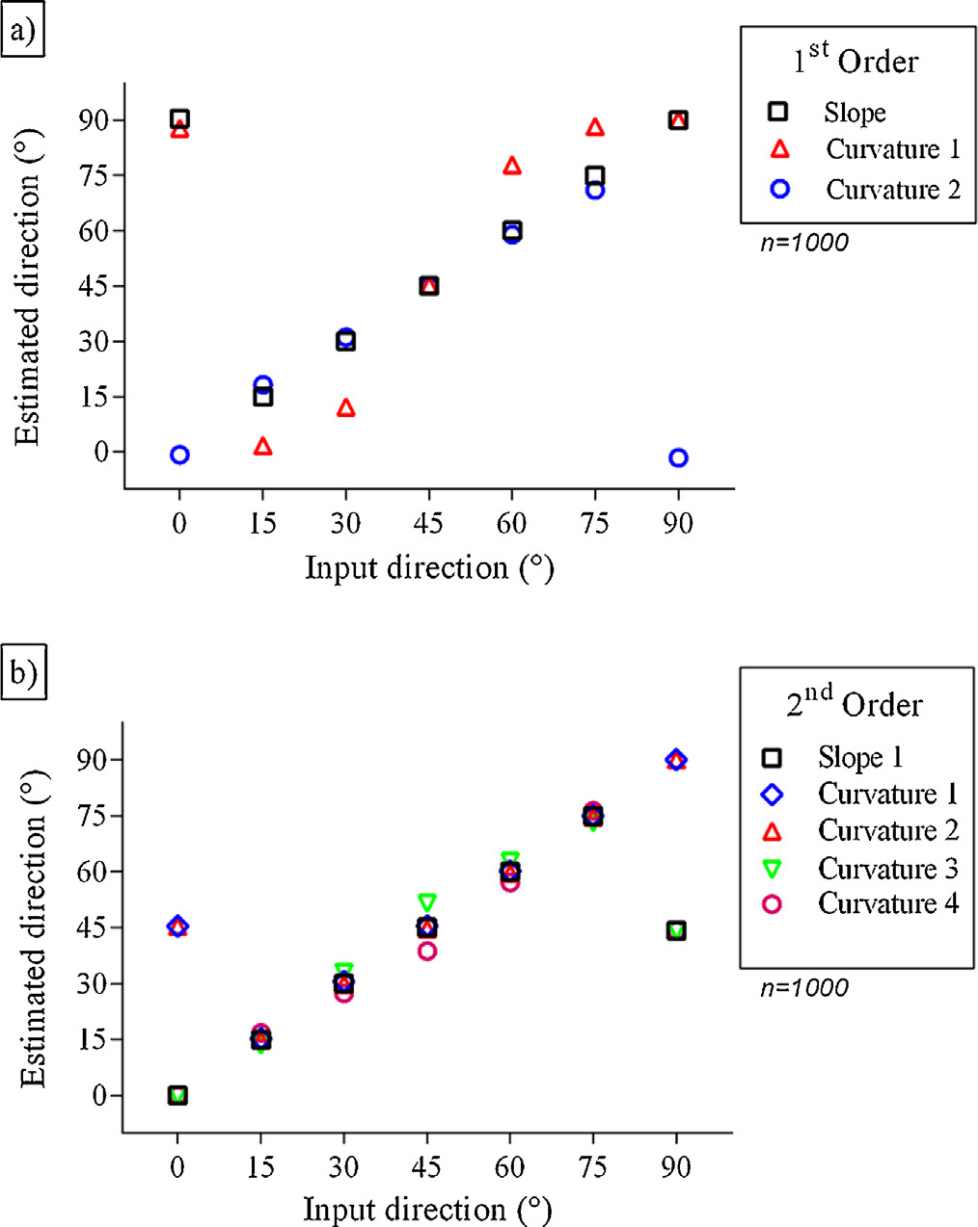
Directions estimated using wave data records calculated by the system with seven different direction inputs and first-order (a) and second-order (b) coefficients.

The first order coefficient calculated by the equation Curvature 1 (Table 2) showed larger differences in direction estimates in relation to the input data of the model (Fig. 2a). The coefficient calculated by Curvature 2 showed good results on direction estimates but with a lag in estimates of waves coming from an angle of 90°. The advantage observed with this coefficient was in estimating direction close to 0°, where there is a lag shown by calculations using the slope parameters.

The second order coefficients, calculated by the Curvature parameters (Table 2), resulted in a mirror image among Curvatures 1–2 and 3–4, with estimates consistent in almost any quadrant (Fig. 2b). Curvatures 1 and 2 were the only equations that showed accurate results for waves from 90°.

**Fig. 3:**
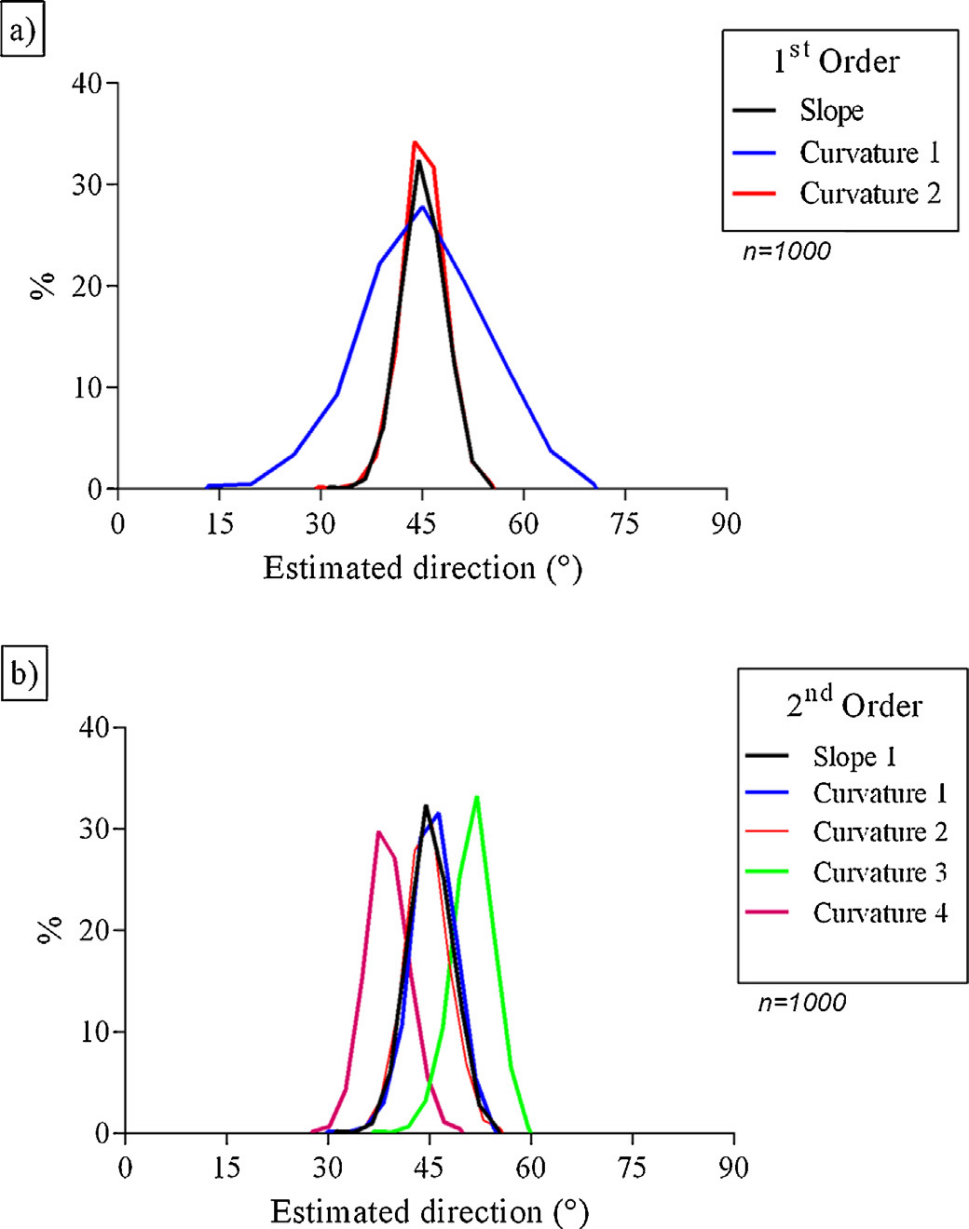
Percentage of means directions estimated by first order (a) and second order (b) of Fourier coefficients (input: 45°).

The directions estimated from the peak frequency (input 45°) are presented in the diagrams of frequency of occurrence (Fig. 3) and showed a tendency for a normal distribution that was observed for all estimates. The wave direction estimated by first order Fourier coefficients (Fig. 3a) obtained from Slope, Curvature 1 and Curvature 2 (Table 2) showed a mean value similar to data input (Table 1) and low values of standard deviation (Table 3). In addition, the wave direction estimated by Curvature 1 had the greatest standard deviation and the Curvature 2 had the lowest.

The wave direction estimated by second order Fourier coefficients obtained from the curvature parameters (Fig. 3b) showed good precision but low accuracy. However, directions estimated from the pair of parameters Curvatures 1–2 and Curvatures 3–4 showed results as a mirror image with a well accurate mean and showed the lowest standard deviation, indicating better precision (Table 3).

## Additional informations

### Background

Several studies have successfully used arrays of sea surface level measurement to estimate directional properties of waves (e.g. [6], [7], [8], [9], [10], [11], [12], [13]). Such studies, however, have not been successful for estimating directions of waves in shallow water environment. Wave direction can be well estimated by first order coefficients of Fourier series in deep waters and the estimates are usually more stable and present the best estimate of wave direction [14], [15], [16], [17]. In shallow waters, however, changes in the system are required to enable adequate estimation of wave direction. This is because different sizes, frequencies and directions of waves intersect at the same time. The curvature provides not only the first order, but also the second order of Fourier coefficients. This allows to better estimate big waves by the first order and small waves by the second order of Fourier coefficients in shallow water. Moreover, wave properties change as they approach the shore; the wave orbit becomes elliptical, more asymmetrical and slender due to friction with the sea floor. Thus, the use of nonlinear terms in wave equations such as curvature should be considered to improve the estimate of wave directions in shallow waters.

To permit the accurate measurement of waves, several wave gauges have been developed to measure the water level, such as include wire resistance, pressure sensors and optical sensors e.g. [18], [19], [20], [21], [22], [23]. The cloverleaf wave meter developed by Cartwright and Smith [24] is the only that proposed to measure the curvature of the wave, but details of the results were not shown [25] and the data are analyzed using acceleration and slope parameters [26], also measured by the cloverleaf. In addition, an aligned array of three wave gauges allows approaching the wave curvature by finite difference adjustment by second order parabolic equations. The curvature allows to increase the number of ways to calculate the Fourier coefficients used to estimate wave direction and it is expected that can improve the estimation of the wave direction in shallow water.

The costs associated with the deployment of a directional wave measurement system are generally very high, considering the technological resources and operational difficulties of installation and maintenance in seawater environments. Thus, the factors that define the quality of wave measurement systems, such as precision, accuracy, resolution, efficiency of processing software and influence of noise on the signal, need to be evaluated before system fabrication and installation. The efficiency of the method, as well as details of the analysis of the wave parameters can be tested before fabrication of wave meter and installation in a fixed structure.

## Figures and Tables

**Table 1:** Data input in the model simulation.

Data input of the model	
Sampling interval (s)	1
Water depth (m)	10
Significant height (m)	0.3
Peak period (s)	3
Minimum frequency (Hz)	0.01
Maximum frequency (Hz)	0.5
Mean propagation direction (°)	45
Maximum propagation direction (°)	60
Minimum propagation direction (°)	30

**Table 2:** Fourier coefficients equation calculated using curvature of the wave.

1st order pair of coefficients
Curvature 1: $a_{1}=-\frac{4Q\eta_{x}\eta_{xx}}{3\pi S(f)k^{3}}$; $b_{1}=-\frac{4Q\eta_{y}\eta_{yy}}{3\pi S(f)k^{3}}$
Curvature 2: $a_{1}=-\frac{4Q\eta_{x}\eta_{yy}}{\pi S(f)k^{3}}$; $b_{1}=-\frac{4Q\eta_{y}\eta_{xx}}{\pi S(f)k^{3}}$

2nd order *a*_2_ coefficients
Curvature 1: $a_{2}=-\frac{2}{\pi }\left[\frac{C\eta \eta_{xx}}{S(f)k^{2}}+\frac{1}{2}\right]$
Curvature 2: $a_{2}=\frac{2}{\pi }\left[\frac{C\eta \eta_{yy}}{S(f)k^{2}}+\frac{1}{2}\right]$
Curvature 3: $a_{2}=-\frac{2}{\pi }\left[\frac{C\eta_{yy}\eta_{yy}}{S(f)k^{4}}-\frac{3}{8}\right]$
Curvature 4: $a_{2}=\frac{2}{\pi }\left[\frac{C\eta_{xx}\eta_{xx}}{S(f)k^{4}}-\frac{3}{8}\right]$

S: autospectra; C: cross-spectra; Q: quad-spectra.

**Table 3:** Mean and standard error of the mean direction estimated from 1000 data records (input: 45°).

	1st order			2nd order					
	Sp.	Cv. 1	Cv. 2	Sp.	Cv. 1	Cv. 2	Cv. 3	Cv. 4	Mean Cv.
Mean (°)	45.09	45.27	45.08	45.08	45.36	44.8	51.23	38.92	45.08
Std. Dev.	3.26	9.09	3.18	3.27	3.30	3.25	3.13	3.16	3.17

Sp.: slope; Cv.: curvature.
