# Supplementary material for: Determining wave direction using curvature parameters
Source: MethodsX. 2016 Jan 16;3:53–61. doi: 10.1016/j.mex.2016.01.003 (PMC4929270; doi:10.1016/j.mex.2016.01.003)
Supplement: Supplementary file 2 [file mmc2.pdf]

Determining wave direction using curvature parameters

*Eduardo V. Queiroz[1], João Luiz B. de Carvalho[1]

[1] Laboratory of Physical Oceanography, University of Vale do Itajaı´, Rua Uruguai,458, Itajaı´-SC 88.302-202, Brazil

*Corresponding Author: queirozev@yahoo.com

Tutorial: Scripts for wave parameters simulation and parametrical analysis

The MatLab files contains all the scripts to simulate the wave parameters (Elevation, slope and curvature), and the scripts to analyse the wave directions of the records simulated.

This folder contains 2 primary scripts. The first script is the file “simulation.m” with 2 functions (corrige.m and fpierson.m). The second script is the file “analysis.m” where 5 functions are used (alisa.m, arctang.m, cortheta.m, espec.m and integral.m).

All files should be in a single folder to be able to run the simulation and direction analysis in MatLab R2014b (8.4.0.150421).

To run the simulation and analyses, follow the subsequent steps:

1- Define in Matlab the folder that includes all the files;

2- Open the file “simulation.m” in Matlab;

3- Once this is open, run the simulation. The output of the simulation (data file of the records of the wave parameters) will be created in the same folder as the simulation file.

4- Open and run the file “analysis.m”. 5 graphs will be created (Energy spectra, number of waves, check ratio, histograms of mean directions estimated by 1st and 2nd order of Fourier coefficients.
